# Prevalence of metabolic syndrome in pre- and postmenopausal women

**DOI:** 10.1590/2359-3997000000253

**Published:** 2017-02-01

**Authors:** Ricardo de Marchi, Cátia Millene Dell’Agnolo, Tiara Cristina Romeiro Lopes, Angela Andréia França Gravena, Marcela de Oliveira Demitto, Sheila Cristina Rocha Brischiliari, Deise Helena Pelloso Borghesan, Maria Dalva de Barros Carvalho, Sandra Marisa Pelloso

**Affiliations:** 1 Departamento de Ciências da Saúde Universidade Estadual de Maringá PR Brasil Departamento de Ciências da Saúde, Pós-Graduação em Ciências da Saúde, Universidade Estadual de Maringá (UEM), PR, Brasil.; 2 Departamento de Enfermagem Universidade Estadual de Maringá PR Brasil Departamento de Enfermagem, Pós-Graduação em Enfermagem, Universidade Estadual de Maringá (UEM), PR, Brasil.

**Keywords:** Metabolic syndrome, menopause, climacteric, cardiovascular disease

## Abstract

**Objective:**

The objective of this study was to determine the prevalence of metabolic syndrome (MS) and its components among pre- and postmenopausal women, as well as the association between menopausal status and MS.

**Materials and methods:**

A retrospective study was conducted at a reference cardiology outpatient clinic in a city located in Northwestern Paraná State, Brazil. A total of 958 medical records of symptomatic climacteric women evaluated between 2010 and 2014 were analyzed. The study consisted of two groups: pre- and post-menopausal women. MS was characterized according to the criteria of the National Cholesterol Education Program’s Adult Treatment Panel III – NCEP-ATP III-2005.

**Results:**

MS was observed in 18.5% of the total study population; 9.4% of the premenopausal women and 22.2% of the postmenopausal women displayed MS, corresponding to a relative risk of 2.75. In addition, the frequency of MS increased with age. Regarding the components of MS, postmenopausal women were more likely to have high density lipoprotein (HDL-C) levels < 50 mg/dL; systolic blood pressure (SBP) values ≥ 130 mmHg or diastolic blood pressure (DBP) values ≥ 85 mmHg; and fasting glucose levels ≥ 100 mg/dL.

**Conclusion:**

MS was more prevalent among postmenopausal women than among premenopausal women.

## INTRODUCTION

Cardiovascular diseases (CVDs) are the leading cause of death among women in the United States. According to the American Heart Association (AHA), approximately one in three female adults has some form of CVD (1).

The cardiovascular risk profile coincides with menopause and it is characterized by the occurrence or worsening of some risk factors associated with this period such as abdominal obesity, hypertension, and dyslipidemia (2,3). These risk factors, in combination with insulin resistance, hyperinsulinemia, and hyperglycemia, compose metabolic syndrome (MS). Patients with MS are at increased risk of CVD and type 2 diabetes mellitus (2).

Postmenopausal status is associated with an increased incidence of MS and CVD, which is mainly associated with the reduction in the levels of female sex hormones (4). Studies have reported that menopause is an independent predictor of MS in females (5,6).

Several studies have identified risk factors for MS and CVD in menopausal women (7,8). However, few studies address this topic in the Brazilian population. Therefore, the objective of this study was to determine the prevalence of MS and its components in pre- and postmenopausal women, as well as the association between menopausal status and MS.

## MATERIALS AND METHODS

This retrospective study was performed in a reference cardiology outpatient clinic in a city located in Northwestern Paraná State, Southern Brazil. A total of 958 medical records of women aged 40-65 years evaluated between 2010 and 2014 were analyzed. All of the women seen in the clinic during the study period were included in the study, except for hysterectomized women.

The study consisted of two groups: a premenopausal group composed of women who were still having either regular or irregular menstrual cycles and a postmenopausal group composed of women who had not experienced menstrual cycles in more than one year, according to the definition of the guideline on the diagnosis and management of menopause (9).

The presence of MS is considered a disorder associated with a set of cardiovascular risk factors including abdominal fat deposition, hypertension, low levels of high density lipoprotein cholesterol (HDL-C), elevated levels of low density lipoprotein cholesterol (LDL-C), hypertriglyceridemia, increased fasting glucose levels diagnosed according to the criteria of the National Cholesterol Education Program’s Adult Treatment Panel III – NCEP-ATP III-2005 (2), and an increased body mass index (BMI) calculated by dividing the body weight (kg) by the height squared (m^2^). The subjects were classified as non-obese (BMI up to 29.9 kg/m^2^) and obese (BMI equal to or greater than 30 kg/m^2^) according to the World Health Organization (WHO) standards (10). According to the NCEP-ATP III, MS represents the combination of three of the following variables (2):

1. Abdominal obesity: waist circumference ≥ 88 cm;
2. Hypertriglyceridemia: serum TG levels ≥ 150 mg/dL;
3. Serum HDL-c, low: < 50 mg/dL;
4. Hypertension: systolic blood pressure (SBP) ≥ 130 mmHg and/or diastolic blood pressure (DBP) ≥ 85 mmHg or receiving treatment for hypertension; and
5. Elevated fasting glucose: glucose level > 100 mg/dL or receiving treatment for diabetes.

The following variables were analyzed: age, stratified into age groups (40-45 years, 46-50 years, 51-55 years, and 56-65 years); color (white and non-white); civil status (with or without a partner); paid occupation (yes or no); physical activity (yes or no); tobacco and alcohol use (yes or no); systolic and diastolic blood pressure values; waist circumference (WC); and an analysis of fasting glucose, HDL-C, LDL-C, and triglycerides levels and the BMI (obese or non-obese).

The values for WC, blood pressure and laboratory tests were obtained from the medical records of the participants. The outpatient clinic uses a standardized procedure for measuring blood pressure; namely, after a 5-min rest, blood pressure is measured in the left arm both at the beginning and end of the examination using an aneroid sphygmomanometer, and the patient is in a seated position. The WC corresponds to the midpoint between the lower margin of the last rib and the top of the iliac crest. The laboratory tests were performed in different laboratories, always after a 12-h fasting period.

For the statistical analysis, a descriptive analysis and a crude analysis were performed using the chi-square test. The risk of MS and its components was analyzed with the crude odds ratio (OR) using the chi-square test. The program Epi Info 3.5.1 was used, and a significance level of 5% and confidence interval (CI) of 95% were adopted.

The research project was approved by the Standing Committee on Research Ethics of the State University of Maringa (Universidade Estadual de Maringá – UEM), under decision number 856.300. A waiver of signed informed consent was requested because the data were obtained from medical records without patient identification.

## RESULTS

The medical records of 958 climacteric women, including 277 premenopausal women (28.9%) and 681 postmenopausal women (71.1%), were analyzed. The overall mean age was 53.6 ± 7.52 years, with a large percentage of women aged 56-65 years (46.0%). The mean ages of the pre- and postmenopausal women were 44.5 ± 2.9 and 57.3 ± 5.33 years, respectively. According to the socio-demographic variables, 71.3% of the women had a partner, 95.4% were white, and 57.4% had a paid occupation.

MS was observed in 18.5% of women. MS was more prevalent among postmenopausal and the oldest women. A total of 9.4% of premenopausal women presented MS, while 22.2% of postmenopausal women presented this syndrome, with a relative risk of 2.75 (CI 1.76-4.28). Regarding the age groups, MS was more frequent with increasing age (Table 1).

**Table 1:** Prevalence of metabolic syndrome according to menopausal status, sociodemographic data, and lifestyle. Sarandi, Paraná, Brazil, 2015

	n	% MS		OR	95% CI	p

		Yes n (%)	No n (%)			
Menopausal status	958					
Premenopausal		26 (9.4)	251 (90.6)	1		
Postmenopausal		151 (22.2)	530 (77.8)	2.75	1.76-4.28	< 0.001
Age group (years)	958					
40-45		10 (5.4)	174 (94.6)	1		
46-50		20 (12.7)	137 (87.3)	2.54	1.15-5.60	0.01
51-55		36 (19.9)	145 (80.1)	4.32	2.07-9.00	< 0.001
56-65		111 (25.5)	325 (74.5)	5.94	3.03-11.64	< 0.001
Skin color	951					
White		166 (18.3)	742 (81.7)	1		
Non-white		9 (20.9)	34 (79.1)	1.18	0.55-2.51	0.66
Marital status	937					
With partner		125 (18.5)	552 (81.5)	1		
Without partner		49 (18.8)	211 (81.2)	1.02	0.71-1.47	0.89
Paid occupation	951					
Yes		92 (16.5)	464 (83.5)	1		
No		84 (21.3)	311 (78.7)	1.36	0.98-1.89	0.06
Physical activity	294					
Yes		24 (16.1)	125 (83.9)	1		
No		21 (14.5)	124 (85.5)	0.88	0.46-1.66	0.69
Tobacco use	581					
Yes		12 (19.0)	51 (81.0)	1.18	0.60-2.31	0.62
No		86 (16.6)	432 (83.4)	1		
Alcohol use	845					
Yes		3 (15.8)	16 (84.2)	0.87	0.25-3.08	1.00
No		82 (17.6)	384 (82.4)	1		

MS: metabolic syndrome; OR: odds ratio; CI: confidence interval.

Table 2 shows the association between the components of MS according to menopausal status. Postmenopausal women were more likely to have HDL-C levels < 50 mg/dL (OR 1.53; CI 1.08-2.18); SBP values ≥ 130 or DBP values ≥ 85 mmHg (OR 2.47; CI 1.85-3.31); and fasting glucose levels ≥ 100 mg/dL (OR 2.04; CI 1.30-3.21).

**Table 2:** Presence of metabolic syndrome and its components according to menopausal status. Sarandi, Paraná, 2015

	N	Premenopause		Postmenopause		OR	95% CI	p
	
		N	%	N	%			
WC ≥ 88 cm	213							
Yes		31	23.3	102	76.7	1.32	0.70-1.49	0.37
No		23	28.8	57	71.3	1		
TGL ≥ 150 mg/dL	673							
Yes		40	21.5	146	78.5	1.44	0.96-3.15	0.07
No		138	28.3	349	71.7	1		
HDL-C < 50 mg/dL	661							
Yes		71	22.2	249	77.8	1.53	1.08-2.18	0.01
No		104	30.5	237	69.5	1		
LDL-C ≥ 130 mg/dL	629							
Yes		57	25.6	166	74.4	1.10	0.74-1.62	0.62
No		111	27.3	295	72.7	1		
SBP ≥ 130 or DBP ≥ 85 mmHg	932							
Yes		134	22.3	468	77.7	2.47	1.85-3.31	< 0.001
No		137	41.5	193	58.5	1		
Fasting glucose levels > 100 mg/dL	501							
Yes		32	17.2	154	82.8	2.04	1.30-3.21	0.001
No		94	29.8	221	70.2	1		
BMI (kg/m^2^)	204							
< 29.9		28	26.7	77	73.3	1		
≥ 30		30	30.3	69	69.7	0.84	0.43-1.61	0.56

WC: waist circumference; TGL: triglycerides; SBP: systolic blood pressure; DBP: diastolic blood pressure; BMI: body mass index; OR: odds ratio; CI: confidence interval.

## DISCUSSION

Few Brazilian studies address the correlation between MS and menopause, which sets the present study apart. However, some study limitations should be considered. Data were obtained from electronic medical records that were entered only once during medical visits and may have been incomplete. In fact, because the present study evaluated medical records, some relevant factors associated with MS were missing, such as lifestyle, physical activity, tobacco use, and obesity. The frequency and intensity of physical activity were not reported. Similarly, the tobacco use data were incomplete, as the time and frequency of use were not collected. Another important limitation of this study is that the sample comprised women who were observed at a reference cardiology outpatient clinic, and thus, they had a greater likelihood of presenting with cardiovascular risk factors and other comorbidities.

In this study, MS was present in 18.5% of the studied women. Another Brazilian study found a higher prevalence (34.7%) of MS in this group of women in the state of Maranhão (11). In studies from different countries, the prevalence of MS in women has ranged from 15.9% in Thai women (12) to 26.4% in Iranian women (5) and 33.8% in *Puerto Rican* women (13).

Our study found that MS was more prevalent among postmenopausal women (22.2%) than among premenopausal women (9.4%). The prevalence of MS among postmenopausal women has been reported to vary by country, ranging between 16.9% in Thailand (12), 29.0% in Puerto Rico (13), 31.0% in Iran (14), 49.8% in Brazil (11), 53.5% in Iran (5), 55.5% in India (15), and 64.3% in Iran (16). MS is one factor that increases the mortality rate in both men and women (17). A meta-analysis confirmed this information by describing a higher prevalence of MS during the postmenopausal stage than the premenopausal stage, regardless of population (18).

There was a statistically significant relationship between postmenopausal stage and increased age with the presence of MS. Older women (older than 56 years of age) were 5.95 (CI: 3.03-11.64) times more likely than younger women to be diagnosed with MS. Some authors have identified age as the main risk factor for increased MS prevalence (10,19). The prevalence of MS varies among studies; however, several Brazilian (11,20) and international studies (15,16,21) found a greater MS prevalence among postmenopausal women than among premenopausal women. A study performed in women aged 40-65 years in Argentina, another South American country, demonstrated a relative risk of 1.61 (CI: 1.18-2.19) of developing MS during post-menopause (22). The Third National Health and Nutrition Examination Survey (NHANES) III study described the association between MS and increased risk of mortality among postmenopausal women compared to premenopausal women (23).

Unfavorable cardiovascular risk factor levels are observed during menopause, including changes in body fat distribution from a gynaecoid pattern to an android pattern, abnormal blood lipid levels, increased sympathetic tone, endothelial dysfunction, vascular inflammation, and increased blood pressure. Postmenopausal women are at greater risk for CVDs than men (matched by age) due to the failure and reduction of the gonads and steroid production (24). Estrogens play a key role in maintaining adequate levels of HDL-C (8).

In our study, the prevalence of all MS components was higher in postmenopausal women, with a statistically significant association for low HDL-c levels, hypertension, and high fasting glucose levels. These components were also described as prevalent in postmenopausal women in Iran (5,6), South Korea (25), and Poland (26), as well as in Brazil (20). A study conducted in the state of Maranhão revealed that increased blood pressure was also prevalent (73.4%) among postmenopausal women (11). However, a recent study found no significance differences for body weight, BMI, WC, blood pressure, total cholesterol, LDL cholesterol, triglycerides and glucose levels between premenopausal and postmenopausal women (27).

Some researchers consider post-menopause as a period of hyperandrogenism that results from the greater reduction in estrogen, due to ovarian failure, than in androgens, with increased levels of LDL and decreased levels of HDL cholesterol, which characterize an atherogenic profile (28,29), compatible with MS. Estrogen seems to have a positive effect on the inner layer of artery wall, which helps to maintain blood vessels flexible (30). Conversely, the cause of hypertension is not well defined in postmenopausal women. It is believed that an increased androgen/estrogen ratio can alter the renin-angiotensin system (31). Other possible causes for hypertension are increased endothelin levels, oxidative stress, obesity and stimulation of the sympathetic nervous system (32). Whether administered chronically, endothelin causes increases in sodium reabsorption in the kidney and consequent increase in blood pressure (33), and in postmenopausal women, plasma endothelin levels are increased (34), suggesting that endothelin can play a part in increasing blood pressure after menopause (35). In conclusion, both endothelin and angiotensin II may contribute to oxidative stress (36). The oxidative stress markers are increased in women after menopause (37), and oxidative stress has caused the increase of blood pressure by decreasing the bioavailability of vasodilator (36). However, antioxidant therapy did not produce a reduction in blood pressure, in humans (38). The role of oxidative stress in hypertension in women after menopause has not been completely elucidated (35).

However, some authors found that although HDL cholesterol levels decrease with increased visceral fat and total weight, low HDL cholesterol levels are not a main feature of MS in postmenopausal women. HDL cholesterol levels appear to increase, not decrease, with age (27). Moreover, higher HDL cholesterol levels were found in postmenopausal women compared to premenopausal women (27,39).

In our study, we found a higher risk of glucose levels > 100 mg/dL. A study conducted with diabetic women found a high prevalence of MS in both premenopausal (87.5%) and postmenopausal (87.7%) women (24). Insulin resistance is described as a key factor implicated in the pathophysiology of MS (40). It contributes to increased glucose intolerance and diabetes, hypertension, increased triglyceride levels, and reduced HDL levels (41).

Another study revealed that postmenopausal women were more likely to present elevated total cholesterol levels, poor glycemic control (OR = 2.92; 95% CI = 1.32-6.33), and lower HDL levels (OR = 0.36; 95% CI = 0.19-0.68) than premenopausal women (42).

Although the BMI was not correlated with menopausal status in the present study, a previous study demonstrated that a BMI > 30 kg/m^2^ (obese subjects) had a significant negative effect on blood pressure (increase), triglycerides, and fasting glucose levels, in addition to being associated with low HDL-C levels compared to a normal BMI (non-obese subjects). These data indicate that “obese” individuals have more cardiovascular risk factors (43).

Finally, some authors report that the prevention of metabolic diseases in menopause requires changes in lifestyle, including the performance of moderate physical activity and consumption of a healthy diet, as the main recommendation to prevent metabolic diseases during menopause. In some cases, after an individual evaluation, hormone replacement therapy was recommended (44), which could have a positive effect on lipids by reducing total and LDL cholesterol and by slightly increasing HDL levels, as demonstrated in a meta-analysis (45).

Thus, our data suggest that the prevalence of MS was higher among postmenopausal women than premenopausal women and increased with increasing age. The components of MS that were prevalent in postmenopausal women included low HDL-C levels (< 50 mg/dL), hypertension (SBP values ≥ 130 mmHg or DBP values ≥ 85 mmHg), and high fasting glucose levels (≥ 100 mg/dL).
